# Differential Inhibitory Actions of Multitargeted Tyrosine Kinase Inhibitors on Different Ionic Current Types in Cardiomyocytes

**DOI:** 10.3390/ijms21051672

**Published:** 2020-02-29

**Authors:** Wei-Ting Chang, Ping-Yen Liu, Kaisen Lee, Yin-Hsun Feng, Sheng-Nan Wu

**Affiliations:** 1 Institute of Clinical Medicine, College of Medicine, National Cheng Kung University, Tainan 70101, Taiwan; cmcvecho2@gmail.com (W.-T.C.); larry@mail.ncku.edu.tw (P.-Y.L.); 2 Division of Cardiovascular Medicine, Chi-Mei Medical Center, Tainan 71004, Taiwan; 3 Department of Biotechnology, Southern Taiwan University of Science and Technology, Tainan 71004, Taiwan; 4 Division of Cardiology, Internal Medicine, College of Medicine, National Cheng Kung University Hospital, Tainan 70401, Taiwan; 5 Department of Physiology, National Cheng Kung University Medical College, Tainan 70101, Taiwan; xu3d93np@gmail.com; 6 Division of Oncology, Chi-Mei Medical Center, Tainan 71004, Taiwan; yinhsun.feng@gmail.com; 7 Institute of Basic Medical Sciences, National Cheng Kung University Medical College, Tainan 70101, Taiwan; 8 Department of Medical Research, China Medical University Hospital, China Medical University, Taichung 40402, Taiwan

**Keywords:** Lapatinib, sorafenib, K^+^ current, action potential, cardiomyocytes

## Abstract

Lapatinib (LAP) and sorafenib (SOR) are multitargeted tyrosine kinase inhibitors (TKIs) with antineoplastic properties. In clinical observations, LAP and SOR may contribute to QTc prolongation, but the detailed mechanism for this has been largely unexplored. In this study, we investigated whether LAP and SOR affect the activities of membrane ion channels. Using a small animal model and primary cardiomyocytes, we studied the impact of LAP and SOR on Na^+^ and K^+^ currents. We found that LAP-induced QTc prolongation in mice was reversed by isoproterenol. LAP or SOR suppressed the amplitude of the slowly activating delayed-rectifier K^+^ current (*I*_K(S)_) in H9c2 cells in a time- and concentration-dependent fashion. The LAP-mediated inhibition of *I*_K(S)_ was reversed by adding isoproterenol or meclofenamic acid, but not by adding diazoxide. The steady-state activation curve of *I*_K(S)_ during exposure to LAP or SOR was shifted toward a less negative potential, with no change in the gating charge required to activate the current. LAP shortened the recovery from *I*_K(S)_ deactivation. As rapid repetitive stimuli, the *I*_K(S)_ amplitude decreased; however; the LAP-induced inhibition of *I*_K(S)_ remained effective. LAP or SOR alone also suppressed inwardly rectifying K^+^ and voltage-gated Na^+^ current in neonatal rat ventricular myocytes. The inhibition of ionic currents during exposure to TKIs could be an important mechanism underlying changes in QTc intervals.

## 1. Introduction

Lapatinib (LAP) and sorafenib (SOR) represent a unique group of multitargeted, active small-molecule tyrosine kinase inhibitors (TKIs) currently used to treat hematological/oncological malignancies. Planning for dose escalation has been considered for their clinical use, as many malignancies are thought to be caused by an aberrant tyrosine kinase function [1,2,3]. However, some TKIs have been reported to cause a significant prolongation of the electrocardiographic QTc interval when used to treat patients with advanced carcinoma [4]. Various agents and conditions that cause prolongation of the QTc interval may predispose an individual to tosade de pointes tachyarrhythmia, which may result in syncope or sudden death [5,6]. However, the ionic mechanisms of TKI actions on heart cells are incompletely understood.

Blocking a slowly activating component of the delayed rectifier K^+^ current (i.e., slowly activating delayed-rectifier K^+^ current, *I*_K(S)_) manifests as a prolongation of the action potential (AP) duration in cardiac myocytes and its electrocardiographic surrogate, the QTc interval [7,8]. The KCNQ1 gene, which is located on chromosome 11, is primarily responsible for this type of K^+^ current [7,9]. One of the channelopathies associated with KCNQ1 gene mutations is long QT syndrome type 1 [6,7,8,9,10]. In contrast, gain-of-function mutations in K_V_7.1 can result in atrial fibrillation, short-QT syndrome, or sudden infant death syndrome [6]. Therefore, the possibility has arisen that in addition to their effects on inhibiting tyrosine kinases, these TKI agents (e.g., LAP or SOR) might be important regulators of ionic channels and their clinically applied doses can be escalated to treat various advanced cancers.

Therefore, upon the clinical observation of TKI-induced QTc prolongation, the aim of this study was to evaluate the possible effects of LAP or SOR on ionic currents in LAP-treated mice, in heart-derived H9c2 cells, and in neonatal rat ventricular myocytes (NRVMs). Our findings will shed light on the mechanism of ion channels in TKI-induced arrhythmia from clinical, animal, and cellular perspectives.

## 2. Results

### 2.1. LAP Caused QTc Prolongation in the Mouse Model

**Figure 1:**
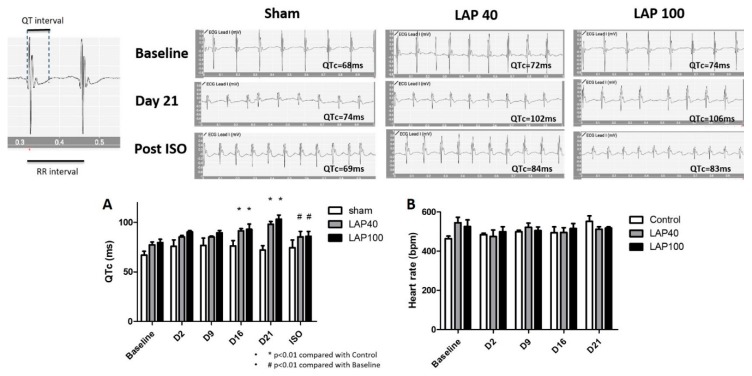
(**Top**) Effects of lapatinib (LAP) on QTc prolongation in the mouse model. (**A**) The changes of QTc prolongation among the sham mice and mice treated with LAP 40 mg/kg/day (LAP 40) and 100mg/kg/day (LAP 100). (**B**) The measurements of the heart rate in mice treated with LAP. * *p* < 0.05 by contrasts from one-way analysis of variance (ANOVA). The *x*-axis in the upper section is given at a standard scale in ECGs.

Compared with the sham group, mice receiving an oral gavage of LAP developed QTc prolongation after 16 days and the changes continued till Day 21 (Figure 1 Top,1A). Generally, the QTc interval extended from seventy to more than one hundred seconds in an LAP dose-dependent manner. To investigate whether isoproterenol, which is an activator of *I*_K(S)_, could reverse the LAP-mediated QTc prolongation, we added isoproterenol (0.17 mg/kg intraperitoneally) after 21 days of LAP treatment and interestingly, the QTc prolongation diminished in both groups of LAP 40 and LAP 100. Additionally, there were no significant changes of the heart rate in mice receiving LAP compared with the sham group (Figure 1B).

### 2.2. Effect of LAP on the Whole-Cell Slowly Activating Delayed-Rectifier K^+^ Current (I_K(S)_) in Differentiated H9c2 Cells

The whole-cell configuration of the patch-clamp technique was used to investigate the possible effects of LAP on ionic currents in H9c2 cells. Initially, the cells were bathed in Ca^2+^-free Tyrode’s solution and the pipette solution contained 3 mM ATP. Each cell was held at the level of −50 mV, and various voltage steps ranging from −50 to −10 mV in 10 mV increments were applied. As shown in Figure S1A, the direction of the K^+^ current became inward, together with the increase in current amplitude, when Ca^2+^-free, Tyrode’s solution was replaced with high-K^+^ (145 mM), Ca^2+^-free solution. As the cell was held at −50 mV, a more positive depolarizing pulse of −30 mV readily elicited the inward currents, which slowly activated within the first 500 msec of the step pulse. Figure S1Ba shows the average current–voltage (*I*–*V*) relationship for the current amplitude measured at the end of the depolarizing pulses in the presence of 5.4 and 145 mM extracellular K^+^_._ Slowly activated K^+^ inward currents with fast deactivation following a long membrane depolarization also became notable following exposure of the cell to high-K^+^ (145 mM) solution. The relationship between membrane potential and the tail current is illustrated in Figure S1Bb. This population of K^+^ currents, which resemble the K_V_7.1-encoded current, has thus been identified as the slowly activating delayed-rectifier K^+^ current (*I*_K(S)_) [7,8].

The response to other known regulators of *I*_K(S)_ was examined in these cells. As shown in Figure S1C, isoproterenol (1 μM) and iloprost (10 μM) increased the *I*_K(S)_ amplitude by about 19% and 24%, respectively, while chromanol 293B (Chrom; 1 μM) suppressed the *I*_K(S)_ amplitude effectively in H9c2 cells [8]. Isoproterenol binds to β-adrenergic receptors in heart cells [11,12] and iloprost is a synthetic analog of prostacyclin. However, neither chlorotoxin (1 μM) nor margatoxin (1 μM) had any effects on *I*_K(S)_ in these cells. Chlorotoxin and margatoxin block Cl^-^ and K_V_1.3 currents, respectively [13]. These results indicate that the *I*_K(S)_ observed in H9c2 cells is sensitive to stimulation by isoproterenol or iloprost and to inhibition by Chrom, but not by chlorotoxin or margatoxin.

### 2.3. Concentration-Dependent Effects of LAP or SOR on I_K(S)_ in H9c2 Cells

**Figure 2:**
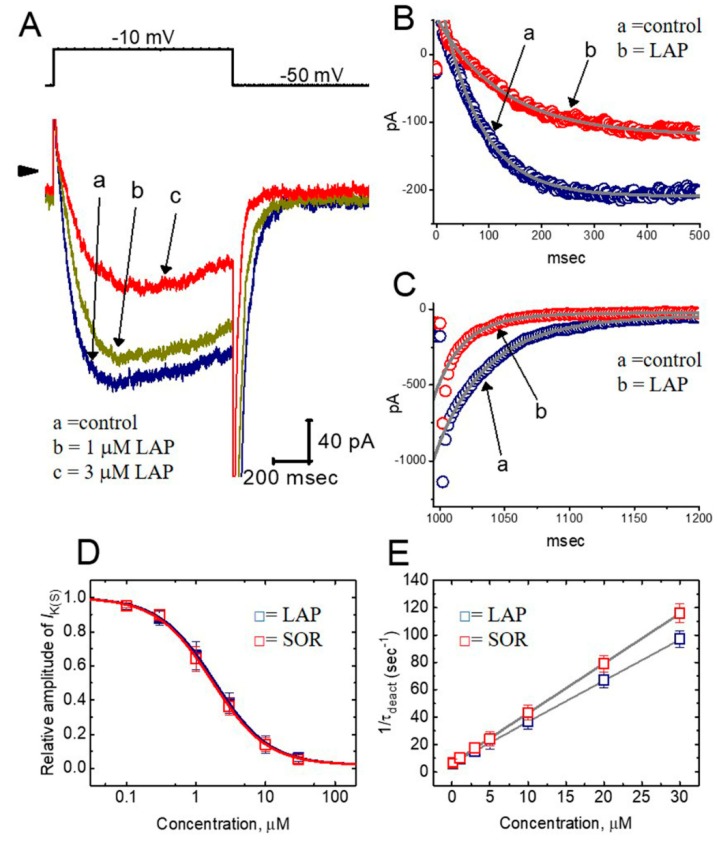
Effect of lapatinib (LAP) or sorafenib (SOR) on the slowly activating delayed-rectifier K^+^ current (*I*_K(S)_) recorded from H9c2 cells. (**A**) Representative *I*_K(S)_ traces elicited by a depolarizing pulse from −50 to −10 mV (indicated in the upper part of (A)). Label ‘a’ is the control and labels ‘b’ and ‘c’ were obtained 2 min after the addition of 1 and 3 μM LAP, respectively. Arrowhead indicates the zero current level. In (**B**) and (**C**), labels ‘a’ and ‘b’ indicate the absence and presence of 3 μM LAP, respectively. The current trajectories shown in (B) were satisfactorily fitted by a single exponential (indicated by the smooth line) with an activation time constant (τ_act_) value of 74.8 msec (a, in the control) and 126.5 msec (b, in the presence of LAP). Those in (C) were fitted by a two-exponential process with the slow and fast deactivation time constant (τ_deact_) values of 90.3 and 35.3 msec (a, in the control) and 60.7 and 31.5 msec (b, in the presence of LAP). (**D**) Concentration-dependent inhibition of *I*_K(S)_ by LAP or SOR (*n* = 8–11 for each point). The relations between the normalized amplitude of *I*_K(S)_ and the concentration of LAP or SOR are illustrated. The values of IC_50_ in the presence of LAP and SOR were 1.84 and 1.69 μM, respectively. (**E**) Evaluation of the kinetics of a LAP- or SOR-induced block of *I*_K(S)_ measured from H9c2 cells. The reciprocal in the slow component of τ_deact_ was plotted versus the LAP (□, blue) or SOR (□, red) concentration (*n* = 8–10 for each point).

The relationship between the LAP or SOR concentration and the percentage inhibition of *I*_K(S)_ was determined. In these experiments, the cells were bathed in a high-K^+^, Ca^2+^-free solution, and the examined cell was depolarized from −50 to −10 mV with a 1 sec duration. Current amplitudes were measured at the end of depolarizing pulses. As illustrated in Figure 2A, LAP or SOR (0.1–30 μM) suppressed the quasi-sustained component of *I*_K(S)_ in a concentration-dependent manner. A nonlinear least-squares fit to the data revealed that the half-maximal concentrations (i.e., IC_50_) required for the inhibitory effect of LAP or SOR were 1.84 and 1.69 μM, respectively, and they almost completely suppressed the *I*_K(S)_ amplitude at a concentration of 30 μM. Therefore, these data indicate that LAP or SOR significantly inhibited the current amplitude in differentiated H9c2 cells.

In addition to the decreased amplitude of *I*_K(S)_, *I*_K(S)_ activation was noted in response to long-lasting depolarization in a time-dependent manner during exposure of the cells to LAP or SOR. In other words, the time course of activating *I*_K(S)_ in the presence of these agents tended to be slowed, while that of deactivating tail current became shortened. The time constants of *I*_K(S)_ activation or deactivation derived from these cells were further analyzed. As depicted in Figure 2B,C, the time courses of *I*_K(S)_ activation and inactivation with or without the addition of LAP were fitted to a single- or two-exponential process. For example, as a depolarizing pulse from −50 to −10 mV was evoked with a duration of 1 sec, LAP (3 μM) significantly increased the activation time constant (τ_act_) required for *I*_K(S)_ elicitation from 74.8 ± 4.1 to 126.5 ± 11.2 msec (*n* = 7, *p* < 0.05). In contrast, the slow component of the deactivation time constant (τ_deact_) for *I*_K(S)_ decreased from 90.3 ± 2.7 to 58.5 ± 2.3 msec (*n* = 7, *p* < 0.05), although no change in the fast component of the *I*_K(S)_ deactivation time constant occurred.

The effects of LAP and SOR on *I*_K(S)_ in response to membrane depolarization were further examined and compared. As shown in Figure 2D, LAP and SOR were effective at suppressing the *I*_K(S)_ amplitude in a concentration-dependent fashion. The concentrations of LAP and SOR required for their effect on the *I*_K(S)_ amplitude measured at the end of the depolarizing pulse were 1.84 and 1.69 μM, respectively. The inhibitory effect of these two compounds on *I*_K(S)_ recorded from H9c2 cells appeared to be similar.

As the *I*_K(S)_, in response to membrane depolarization, tended to increase the deactivating time course of *I*_K(S)_, it would be pertinent to gain information about the kinetics of an LAP- or SOR-induced blockade of these currents measured from H9c2 cells. As shown in Figure 2E, increasing the LAP or SOR concentration not only reduced the steady-state amplitude of *I*_K(S)_, but also enhanced the slow component of the current deactivation in response to membrane depolarization. Based on the minimal binding scheme (see Materials and Methods for details), the estimated *K*_D_ (*k*_−1_/*k*_+1_ *[B]) values in the presence of LAP or SOR were 2.08 and 1.73 μM, respectively, which were similar to the IC_50_ values required to suppress the *I*_K(S)_ amplitude (Figure 2D). Therefore, the inhibitory effect of LAP or SOR on *I*_K(S)_ in H9c2 cells can be presumably explained by a state-dependent blocker that preferentially binds to the open state of the *I*_K(S)_ channel.

### 2.4. Comparisons of the Effects of SOR, LAP, LAP Plus Isoproterenol, LAP Plus Meclofenamic Acid, and LAP Plus diazoxide on the I_K(S)_ Amplitude

The effects of SOR, LAP, LAP plus isoproterenol, LAP plus meclofenamic acid, LAP plus meclofenamic acid, and LAP plus diazoxide on the *I*_K(S)_ amplitude in H9c2 cells were examined and compared. As shown in Figure S2, the further addition of either isoproterenol (1 μM) or meclofenamic acid (10 μM) in the continuous presence of LAP (3 μM) reversed the LAP-induced suppression of the *I*_K(S)_ amplitude, while the subsequent application of diazoxide (30 μM), which is an activator of ATP-sensitive K^+^ channels [14], did not have any effect on the LAP-induced inhibition of *I*_K(S)_. Meclofenamic acid is a known activator of the KCNQ-encoded channel [15]. Moreover, as H9c2 cells were transfected with KCNH2 siRNAs, the reduction of the *I*_K(S)_ amplitude caused by LAP or SOR remained unaltered (data not shown). These results indicate that, within 1 min of exposing H9c2 cells to these agents, there was a decreased *I*_K(S)_, which was not associated with its suppression of *I*_K(erg)_.

### 2.5. Effect of LAP on the I_K(S)_ I–V Relationship in H9c2 Cells

We further examined the effect of LAP on the *I*_K(S)_ amplitude elicited by different levels of depolarizing pulses. Figure S3A shows that *I*_K(S)_ was evoked by various step pulses with a duration of 1 sec, before and after applying LAP (3 μM). As H9c2 cells were exposed to LAP, the amplitude of inward *I*_K(S)_ was suppressed at voltages of −30 to −10 mV. After LAP was removed, the *I*_K(S)_ amplitude almost returned to the control level. The average *I*–*V* relationships for the current amplitude with or without the addition of LAP are shown in Figure S3B. Specifically, exposing the cells to LAP significantly increased the slope of the linear fit of the *I*_K(S)_ amplitude to the voltages between −30 and −10 mV, namely, whole-cell conductance of *I*_K(S)_, from 10.2 ± 1.1 to 2.9 ± 0.6 nS (*n* = 10, *p* < 0.05). Therefore, these data indicate that the *I*–*V* relationship of *I*_K(S)_ was modified when the cells were exposed to LAP.

Figure S3C shows the *I*_K(S)_ activation curve obtained with or without LAP (3 μM). The plot of relative *I*_K(S)_ amplitude as a function of membrane potential was constructed and fitted with a Boltzmann function. In controls, V_1/2_ = −21.2 ± 1.8 mV and *q* = 8.6 ± 0.6 *e* (*n* = 9), whereas, in the presence of LAP (3 μM), V_1/2_ = −13.9 ± 1.4 mV and *q* = 8.4 ± 0.7 *e* (*n* = 9). These data show that the *I*_K(S)_ activation curve was shifted along the voltage axis to a less negative potential by approximately 7 mV. However, no significant change in the gating charge was demonstrated in the presence of this compound. Therefore, the presence of LAP (3 μM) in H9c2 cells was capable of shifting the *I*_K(S)_ activation curve to less negative potentials with no discernible change in the elementary charge for current activation. Similar results were also observed in cells exposed to 3 μM SOR.

### 2.6. Effect of LAP on the Recovery from I_K(S)_ Deactivation in H9c2 Cells

The dependence of *I*_K(S)_ on the pulse duration with or without LAP was examined. As the interval of the depolarizing pulse from −50 to −10 mV was prolonged, the amplitude of the deactivating tail current increased progressively. The normalized peak current was plotted as a function of pulse duration (Figure S4). In the control, the inward tail current following repolarization to −50 mV was observed by durations as short as 100 msec and tended to saturate when the pulse duration was about 1400 msec. However, the time course for current recovery decreased significantly as cells were exposed to 3 μM LAP. The recovery time constants in the absence and presence of 3 μM LAP were 368 ± 18 and 245 ± 12 msec (*n* = 8), respectively. These data indicate that adding LAP significantly shortened the recovery from the deactivation of *I*_K(S)_ detected in these cells.

### 2.7. Inhibitory Effect of LAP on I_K(S)_ Elicited by a Train of Rapid Repetitive Depolarizations

Mutations in gain- or loss-of-function of the *I*_K(S)_ channel are linked to the occurrence of atrial fibrillation and the *I*_K(S)_ channels have been described to be inherently present in atrial myocytes [16]. Therefore, in another set of experiments, we determined whether the *I*_K(S)_ amplitude induced by a train of repetitive depolarizing stimuli could be altered and whether LAP has any effect on the current amplitude in response to such a maneuver. Under control conditions, a single 1-sec depolarizing step to −10 mV from a holding potential of −50 mV produced a considerably large amplitude with a slowly activating property. Interestingly, the current amplitude decreased progressively as the *I*_K(S)_ was evoked by a train of repetitive stimuli at 50 or 100 Hz (Figure S5A). However, under such a train of repetitive depolarizations used to mimic atrial fibrillation, adding LAP (3 μM) was also effective at suppressing the *I*_K(S)_ amplitude (Figure S5B).

### 2.8. Effect of LAP in erg-Mediated K^+^ Current (I_K(erg)_) in H9c2 Cells

**Figure 3:**
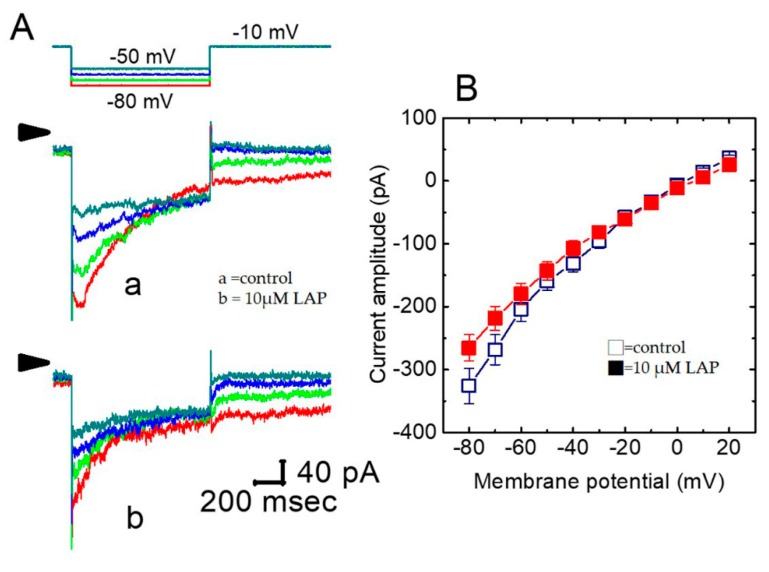
Effect of LAP on *erg*-mediated K^+^ current (*I*_K(erg)_) in H9c2 cells. (**A**) Superimposed current traces obtained when the cell was 1-sec hyperpolarized from −10 mV to various potentials, as indicated in the uppermost part. Current traces labeled ‘a’ are the controls and those labeled ‘b’ were obtained in the presence of 10 μM LAP. The different colors depicted in current traces of ‘a’ and ‘b’ correspond to those in the voltage protocol applied (the uppermost part). (**B**) Averaged *I*–*V* relationships for the peak amplitude of deactivating *I*_K(erg)_ in the absence (□) and presence (■) of 10 μM LAP (*n* = 9–10 for each point). Current amplitudes were measured at the beginning of each hyperpolarizing pulse.

QTc prolongation can also arise from inhibiting another type of K^+^ current, namely, *I*_K(erg)_, which is inherent in heart cells^16^. We also studied whether LAP has any effect on *I*_K(erg)_ recorded from H9c2 cells. In these experiments, the cells were bathed in a symmetrical K-rich concentration (145 mM); the holding potential was set to −10 mV, and a series of hyperpolarizing steps were used to elicit deactivating *I*_K(erg)_. LAP at a concentration of 1 or 3 μM did not produce any effect on the *I*_K(erg)_ amplitude. However, as shown in Figure 3A, adding 10 μM LAP significantly suppressed the current amplitude. For example, as the cells were exposed to 10 μM LAP, the peak amplitude of deactivating *I*_K(erg)_ elicited by membrane hyperpolarization from −10 to −80 mV decreased from 326 ± 28 to 266 ± 21 pA (*n* = 9, *p* < 0.05). Figure 3B depicts the peak amplitude *I*–*V* relationships of deactivating *I*_K(erg)_ with or without 10 μM LAP. However, in the continuous presence of 10 μM LAP, the subsequent application of 10 μM PD-118057, which is an activator of *I*_K(erg)_, was effective in reversing the LAP-mediated inhibition of *I*_K(erg)_. Therefore, the *I*_K(erg)_ is subject to inhibition by LAP to an extent less than that observed for *I*_K(S)_.

### 2.9. Suppressive Effect of LAP on the Amplitude of Inwardly-Rectifying K^+^ Current (I_K(IR)_) Measured from Cultured NRVMs

**Figure 4:**
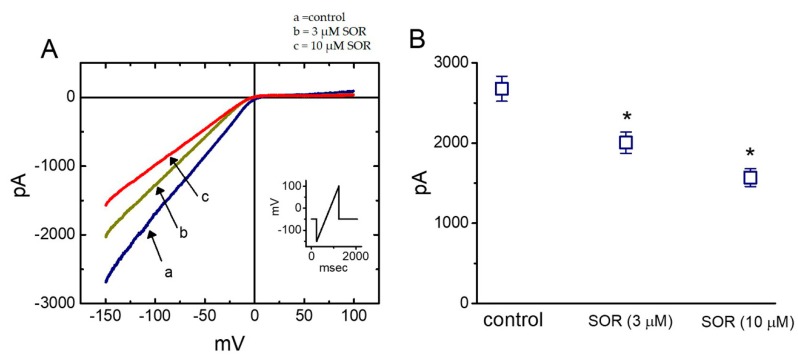
Inhibitory effect of SOR on inwardly-rectifying K^+^ current (*I*_K(IR)_) in neonatal rat ventricular myocytes (NRVMs). (**A**) Superimposed *I*_K(IR)_ traces obtained during the control (a) and in the presence of 3 μM SOR (b) and 10 μM SOR (c). Inset indicates the ramp voltage protocol used. (**B**) Bar graph showing the effect of SOR (3 and 10 μM) on the *I*_K(IR)_ amplitude measured at the level of −150 mV (*n* = 9). * Significantly different from the control, *p* < 0.05 by contrasts from one-way analysis of variance (ANOVA).

In another set of experiments, we explored whether LAP had any effect on *I*_K(IR)_ in heart cells, as modifying *I*_K(IR)_ can influence changes in cardiac Aps [17]. Under our experimental conditions, NRVMs were bathed in a high-K^+^ solution, and the recording pipette was filled with a K^+^-containing solution. Once whole-cell current recordings were made, the cells were held at −50 mV, and a long-lasting ramp pulse from −150 to +100 mV with a duration of 1 sec was applied. As depicted in Figure 4, 3 and 10 μM LAP significantly suppressed the *I*_K(IR)_ amplitude at −150 mV by 25 ± 3% and 41 ± 4% (*n* = 9), respectively. SOR at a concentration of 10 μM also suppressed the *I*_K(IR)_ amplitude. Conversely, LAP or SOR at the same concentration did not significantly affect the current amplitude at the level of +50 mV. Therefore, similar to *I*_K(erg)_ in H9c2 cells, the *I*_K(IR)_ in NRVMs is relatively resistant to inhibition by LAP or SOR.

### 2.10. Effect of LAP on Voltage-Gated Na^+^ Current (I_Na_) in Cultured NRVMs

**Figure 5:**
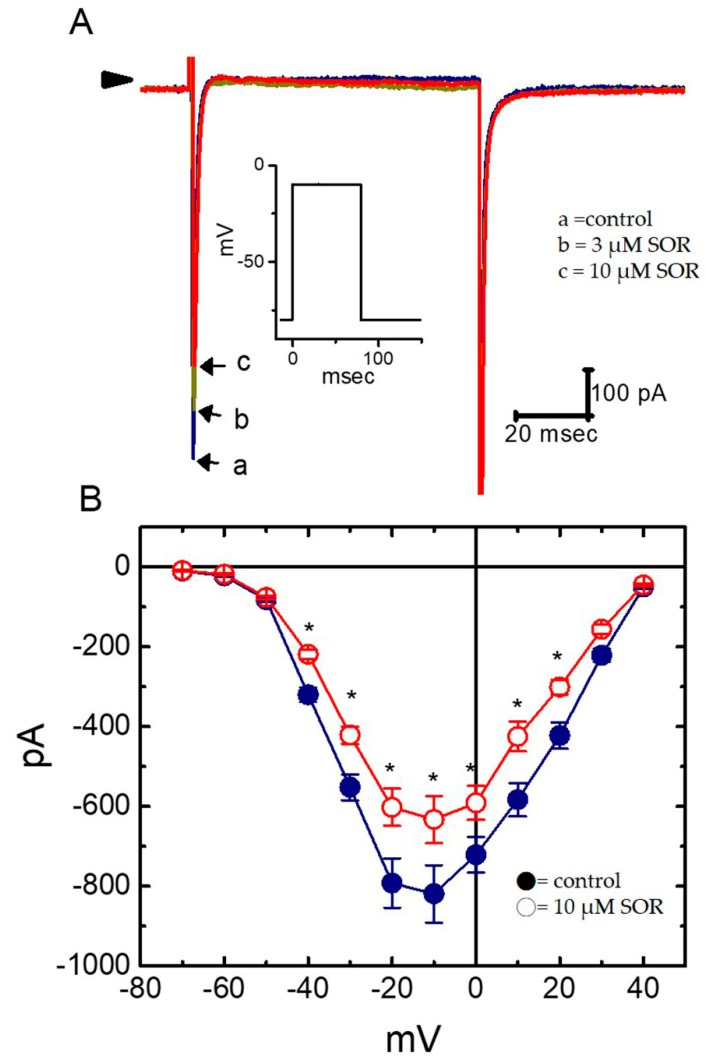
Inhibitory effect of SOR on the voltage-gated Na^+^ current (*I*_Na_) in NRVMs. (**A**) Superimposed *I*_Na_ traces when a cell was depolarized from −80 to −10 mV (indicated in the inset). Label ‘a’ is the control and label ‘b’ and ‘c’ were obtained 2 min after the application of 3 and 10 μM SOR, respectively. Arrowhead indicates the zero current level, and the calibration mark applies to all current traces. (**B**) Averaged *I*–*V* relationships of the peak *I*_Na_ obtained in the absence (●) and presence (○) of 10 μM SOR (*n* = 8–10 for each point). The current amplitudes were measured at the beginning of each depolarizing pulse. * Significantly different from controls (*p* < 0.05).

We also investigated whether LAP perturbs *I*_Na_ in these cells. The cells were bathed in Ca^2+^-free Tyrode’s solution, and the pipette was filled with a Cs^+^-containing solution. LAP (1 μM) had a minimal effect on the peak *I*_Na_ amplitude. However, as shown in Figure 5, 3 and 10 μM LAP significantly suppressed the peak *I*_Na_ elicited by rapid membrane depolarization. For example, as the cell was depolarized from −80 to −10 mV, adding 10 μM LAP significantly decreased the peak *I*_Na_ from 819 ± 72 to 633 ± 59 pA (*n* = 8, *p* < 0.05). After washout of the agent, *I*_Na_ returned to 713 ± 61 pA (*n* = 7). However, the overall *I*–*V* configuration of peak *I*_Na_ did not differ between the absence and presence of LAP (10 µM). Moreover, neither the activation nor the inactivation time course of *I*_Na_ was modified in response to membrane depolarization in the presence of LAP. Similarly, SOR exerted a suppressive effect on peak *I*_Na_. Therefore, LAP or SOR at a relatively high concentration exerted a depressant action on the peak *I*_Na_ in NRVMs.

### 2.11. Effect of LAP on the Membrane Potential Recorded from Cultured NRVMs

**Figure 6:**
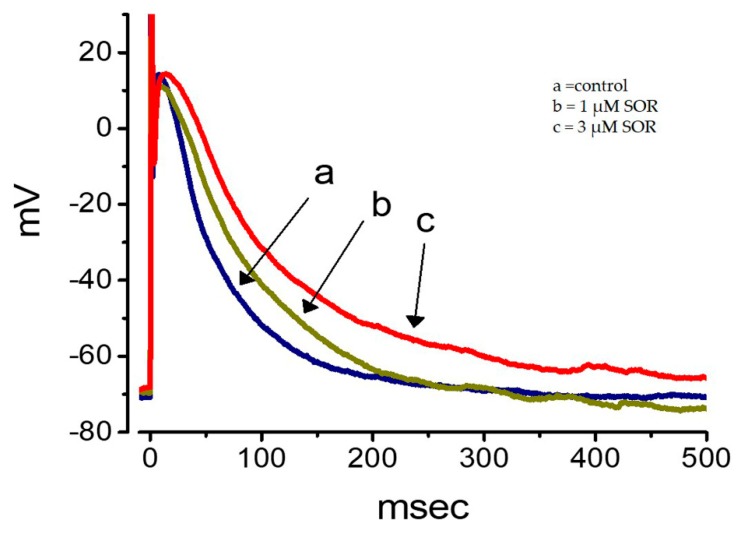
Effect of SOR on the membrane potential in cultured NRVMs. Current-clamp potential recordings were made and cells were bathed in normal Tyrode’s solution containing 1.8 mM CaCl_2_. Potential trace labeled ‘a’ is the control, and those labeled ‘b’ and ‘c’ were obtained during the exposure to 1 and 3 μM SOR, respectively.

In a final set of experiments, we studied whether a TKI (e.g., LAP) has any effects on changes in the membrane potential recorded from NRVMs. As shown in Figure 6, as cells were exposed to 3 and 10 μM LAP, the AP was progressively prolonged, together with slight depolarization of the resting potential. For example, the APD_90_ value in the presence of 10 μM LAP increased significantly to 303 ± 18 msec from the control value of 112 ± 11 msec (*n* = 7, *p* < 0.05). SOR (3 and 10 µM) also prolonged the AP duration to a similar magnitude. These results reflect that LAP- or SOR-mediated lengthening of the cardiac AP tended to be independent of the inhibition of tyrosine kinase and could largely be ascribed to the suppression of transmembrane K^+^ currents.

## 3. Discussion

In this study, we found that LAP or SOR was able to suppress *I*_K(S)_, *I*_K(erg)_, and *I*_K(IR)_ with different potencies; they also suppressed the amplitude of peak *I*_Na_. LAP-induced AP prolongation in cultured NRVMs and this effect was present as QTc prolongation in mice treated with LAP. These effects tended not to be associated with the inhibition of tyrosine kinases and may summate to modify cardiac APs or ECGs caused by these compounds. Our findings explained the phenomenon of patients presenting with QTc prolongation and even subsequent fatal arrhythmia after the treatment of LAP or SOR.

The heart-derived H9c2 cell line, which was established from embryonic rat cardiac ventricles, possesses electrical properties similar to neonatal or developing heart cells. These cells possess a K_V_7.1-type *I*_K(S)_, known as the K_V_7.1 (or KCNQ1)-cloned K^+^ channel [18], which exhibits unique gating properties and voltage dependency, as described in this study, and was thought to be an important contributor to *I*_K(S)_ in H9c2 cells. Therefore, this cell line is a useful model with which to explore the biophysical and pharmacological properties of the *I*_K(S)_ (K_V_7.1) channel.

Perhaps more important than the issue of the magnitude of the LAP- or SOR-induced decrease in *I*_K(S)_ amplitude is that blocking *I*_K(S)_ by these two compounds in response to long-lasting membrane depolarization tends not to be instantaneous, but develops with time, after the channels are opened. Consequently, this effect produced an apparent slowing in the activation and an increase in the deactivation of the current. Therefore, it is possible that the blocking site of LAP or SOR could be time-dependent and located near or within the *I*_K(S)_-channel pore, only when the channel opens, although the detailed mechanism of their loss-of-function actions on *I*_K(S)_ remains to be delineated.

Recent reports have demonstrated that LAP and SOR suppress the proliferation of hepatocellular carcinoma cells by depleting the intracellular ATP content [19]. However, in our experimental whole-cell recordings, the recording pipette was filled with a solution containing 3 mM ATP, which completely suppresses the activity of ATP-sensitive K^+^ channels [6]. As ATP was removed from the internal solution, the *I*_K(S)_ in the examined cell remained functionally active and remained sensitive to suppression by LAP or SOR. Moreover, in the continuous presence of LAP, adding diazoxide failed to reverse the LAP- or SOR-mediated inhibition of *I*_K(S)_. It thus seems unlikely that the LAP- or SOR-mediated inhibition of *I*_K(S)_ observed in H9c2 cells was primarily associated with a decrease in intracellular ATP concentration.

In this study, LAP or SOR suppressed the *I*_K(S)_ amplitude with an IC_50_ value of 1.84 or 1.69 μM, respectively. Similarly, based on the minimal scheme, the estimated *K*_D_ values required for the LAP- or SOR-induced blockage of *I*_K(S)_ were 2.08 and 1.73 μM, respectively. After oral administration was increased from 1750 to 7000 mg per day in the first six cohorts, the plasma LAP concentration was reported to reach 2000–14,000 ng/mL (i.e., 3.4 and 24.1 μM) [20]. The IC_50_ or K_D_ values of LAP or SOR observed in this study were virtually comparable to the plasma concentrations of these drugs used clinically, particularly for the dose escalating profile. Therefore, the inhibitory effect of LAP or SOR on ionic currents presented herein may conceivably occur at a concentration achievable in humans. These data also led us to suggest that isoproterenol, meclofenamic acid, or iloprost may be beneficial for reversing the inhibition of *I*_K(S)_ caused by TKIs.

The concentration of LAP or SOR required for the inhibition of ionic currents appears to be higher than that used to suppress the activity of tyrosine kinase. However, the modifications by LAP or SOR of ionic currents demonstrated herein tend to be acute in onset, and they could hence be independent of their interaction with the activity of tyrosine kinase. The action of these compounds on the amplitude and gating of *I*_K__(S)_ is thought to occur through their preferential binding to the open state of the KCNQ1 channel.

In our study, the presence of LAP or SOR not only reduced the maximal conductance of *I*_K(S)_, but also produced a rightward shift in the steady-state activation curve, although no change in the gating charge for channel activation was demonstrated. The recovery time course of *I*_K(S)_ deactivation was also shortened in the presence of LAP. These results indicate that the observed magnitude of an LAP- or SOR-induced block of *I*_K(S)_ is voltage-dependent and can be altered by different membrane potential levels. The *I*_K(S)_ amplitude reduced by a train of repetitive depolarizations was also blocked by LAP or SOR. As a result, the sensitivity of heart cells to these TKIs would be dependent on the pre-existing resting potential level, the heart rate, or the TKI concentrations achieved, assuming that the in vivo action is the same as those on heart cells shown in this study.

The *I*_K(S)_ (K_V_7.1) channel adopts the classic six transmembrane topology for voltage-gated ion channels and has a large 300-residue intracellular C-terminus important for tetramerization and trafficking [7,9]. The electrical properties of *I*_K(S)_ are dramatically altered by association with different KCNE accessory proteins (KCNE1–KCNE5) [7,9]. The K_V_7.1-KCNE1 complex mediates the *I*_K(S)_ current of the cardiac AP. To what extent TKIs may modify the function of KCNE accessory proteins and alter the magnitude of *I*_K(S)_ remains to be further studied. Conversely, *I*_K(erg)_ or *I*_K(IR)_ was relatively insensitive to blockage by LAP or SOR. As a selective inhibitor of *I*_K(S)_, LAP, SOR, or other structurally similar compounds (e.g., gefitinib) could be a group of valuable pharmacological probes for gaining insight into the possible mechanisms controlling the kinetics and gating of the *I*_K(S)_ (K_V_7.1) channels, because the pore region of the channel protein appears to be of particular relevance for open-channel blockades.

The *I*_K(S)_ is functionally expressed in several other organs or systems. It is thus tempting to speculate that some extra cardiac effects [21] might occur because of the *I*_K(S)_ block caused by LAP or SOR. As the cardiac repolarization reserve is delicately regulated by the magnitude of *I*_K(IR)_ [22], the prolongation of cardiac action potential present in NRVMs could be partly explained by effects on *I*_K(IR)_ produced by LAP or SOR. Moreover, because LAP or SOR produced a conceivable lengthening in cardiac action potential, as well as in the QTc interval of ECG tracing, to what extent these compounds could block the amplitude of transient K^+^ outward currents expressed in heart cells remains to be further examined.

## 4. Materials and Methods

### 4.1. Experimental Animals

Male 12-week-old C57BL/6 mice were obtained from the Animal Center of National Cheng Kung University Medical College. The animal experiments were approved and conducted according to the local institutional guidelines for the care and use of laboratory animals in Chi-Mei Medical Center (approval reference number 103121518; permission number: 100; Date: 10 May 2018) and conformed to the Guide for the Care and Use of Laboratory Animals published by the US NIH (NIH Publication No 85-23, revised 2011). Anesthesia and euthanasia were performed with isoflurane. To evaluate the effect of LAP on myocardial electrophysiology, LAP solved in 0.1% tween 80 + 0.5% hydroxypropyl methylcellulose was administered orally at the doses of 40mg/kg/day (LAP 40) and 100mg/kg/day (LAP 100) (*n* = 4 respectively). Compared with the sham group, both ECG and echocardiography were performed for the sequential three weeks post induction.

### 4.2. ECG Recording and QT Specification in Mice

ECG recordings were performed using an implantable IX-TA-220 iWorx system. Mice under light inhaled anesthesia (2% isoflurane/O_2_). After hair removal, four limbs of the studied mice were contacted to the transmitter device to obtain an approximate lead II, and the heart rate was maintained above 500 beats/min. ECG recordings were collected continuously for ten minutes and only sinus rhythms were analyzed. The QT duration was defined as the interval between the first deviation from the Q wave till the return of the ventricular repolarization to the isoelectric baseline from lead II ECGs. According to Bazett’s formula, each QT was corrected to its own RR interval to obtain the QTc interval.

### 4.3. Isolation and Culture of NRVMs

The cells were isolated from 1- and 2-day-old Sprague-Dawley rats by enzymatic digestion with 0.1% trypsin and 0.03% collagenase, as described previously [6]. After isolation, the cells were plated onto laminin-coated 35 mm dishes at a density of 1 × 10^3^ cells/mm^2^ and cultured for 48 h prior to further experiments.

### 4.4. Electrophysiological Measurements

The cells were dissociated just prior to each experiment, and an aliquot of cell suspension was taken to a home-made recording chamber positioned on the stage of a DM-IL inverted microscope (Leica, Wetzlar, Germany). H9c2 cells or NRVMs were bathed at room temperature (20–25 °C) in normal Tyrode’s solution. Electrophysiological measurements are detailed in the supplementary material.

### 4.5. Statistical Analyses

Values are provided as the mean ± standard error of the mean (SEM), with sample sizes (*n*) indicating the number of cells from which the data were taken; error bars are plotted as SEM. Statistical analyses are detailed in the supplementary material.

## 5. Conclusions

Collectively, the synergistic suppression of *I*_K(S)_, *I*_K(erg)_, and *I*_K(IR)_ caused by LAP or SOR, along with increases in APD, may potentially account for their direct actions on membrane excitability in heart cells and the similar findings obtained from our animal models support this.

## Supplementary Materials

Supplementary materials can be found at https://www.mdpi.com/1422-0067/21/5/1672/s1.

## Abbreviations

ANOVA	analysis of variance
AP	action potential
Chrom	chromanol 293B
I-V	current versus voltage
IC_50_	concentration required for 50% inhibition
*I*_K(erg)_	*erg*-mediated K^+^ current
*I*_K(IR)_	inwardly-rectifying K^+^ current
*I*_K(S)_	slowly activating delayed-rectifier K^+^ current
LAP	lapatinib
NRVMs	neonatal rat ventricular myocytes
QTc interval	corrected QT interval
SOR	sorafenib
τ_act_	activating time constant
τ_deact_	deactivating time constant
TKIs	tyrosine kinase inhibitors
