# Supplementary material for: Differential Inhibitory Actions of Multitargeted Tyrosine Kinase Inhibitors on Different Ionic Current Types in Cardiomyocytes
Source: Int J Mol Sci. 2020 Feb 29;21(5):1672. doi: 10.3390/ijms21051672 (PMC7084345; doi:10.3390/ijms21051672)
Supplement: Supplementary file 1 [file ijms-21-01672-s001.pdf]

International Journal of
*Molecular Sciences*

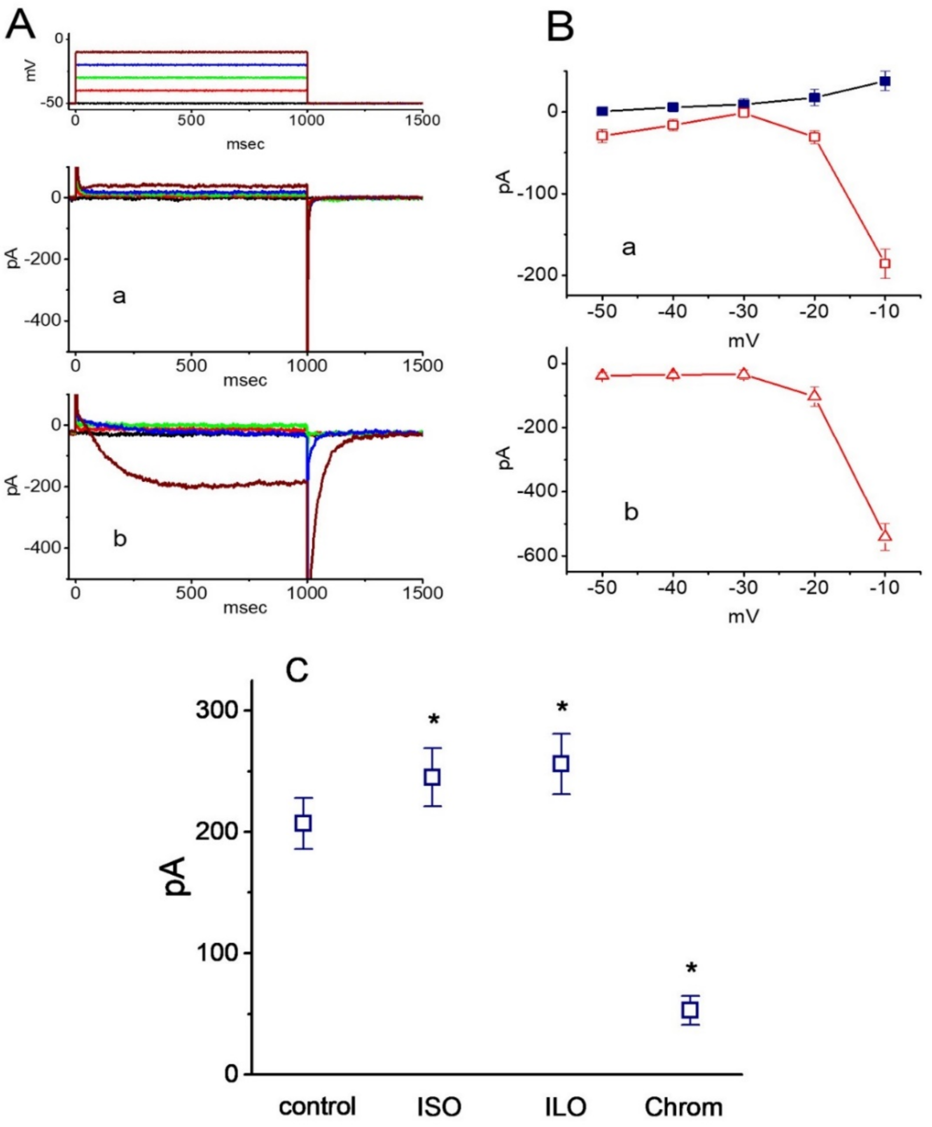


**Figure S1.** Effect of high $K^+$ solution on $K^+$ current in H9c2 cells. The superimposed current traces shown in (**Aa**) are control, and those in (**Ab**) were obtained 2 min after cells were exposed to high-$K^+$ (145 mM), $Ca^{2+}$-free solution. (**Ba**) Averaged current-voltage (*I-V*) relationships of $K^+$ current measured at the end of voltage pulses in the presence of 5.4 mM KCl (filled squares) or 145 mM KCl (open squares) (n=8-12). (**Bb**) Averaged *I-V* relationship for the activation of $I_{tail}$ measured from cells bathed in high-$K^+$, $Ca^{2+}$-free solution (n=8-11 for each point). (**C**) Plot graph showing effect of isoproterenol, iloprost, chromanol 293B on $I_{K(S)}$ amplitude. ($n$ = 7–9 for each point). ISO: 1 μM isoproterenol; ILO: 10 μM iloprost; Chrom: 1 μM chromanol 293B. *Significantly different from control (i.e., high-$K^+$, $Ca^{2+}$-free solution without addition of any agent), $p < 0.05$ by contrasts from one-way analysis of variance (ANOVA).

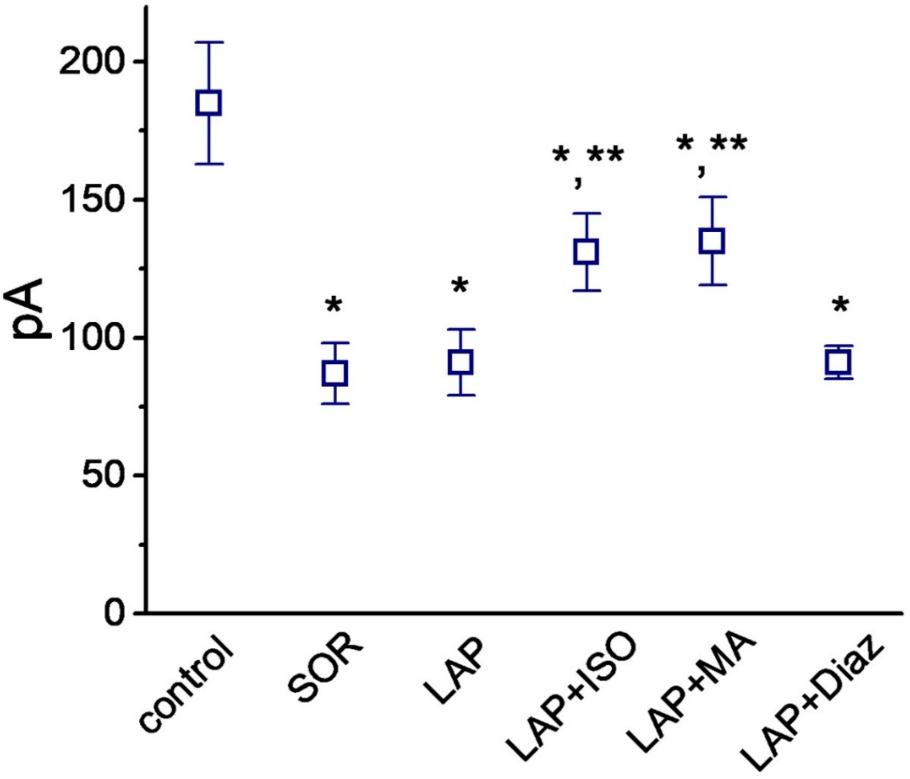


**Figure S2.** Summary of plot graph showing the effects of SOR, LAP, LAP plus isoproterenol, LAP plus meclofenamic acid and LAP plus diazoxide on $I_{K(S)}$ amplitude. Each bar represents the mean±SEM ($n$ = 8–11). SOR: 3 μM SOR; LAP: 3 μM LAP; ISO: 1 μM isoproterenol; MA: 10 μM meclofenamic acid; Diaz: 30 μM diazoxide. (an ATP-sensitive potassium *channel* activator) $^{*}$Significantly different from control (P<0.05) and $^{**}$significantly different from 3 μM LAP alone group ($p < 0.05$). *$p < 0.05$ by contrasts from one-way analysis of variance (ANOVA).

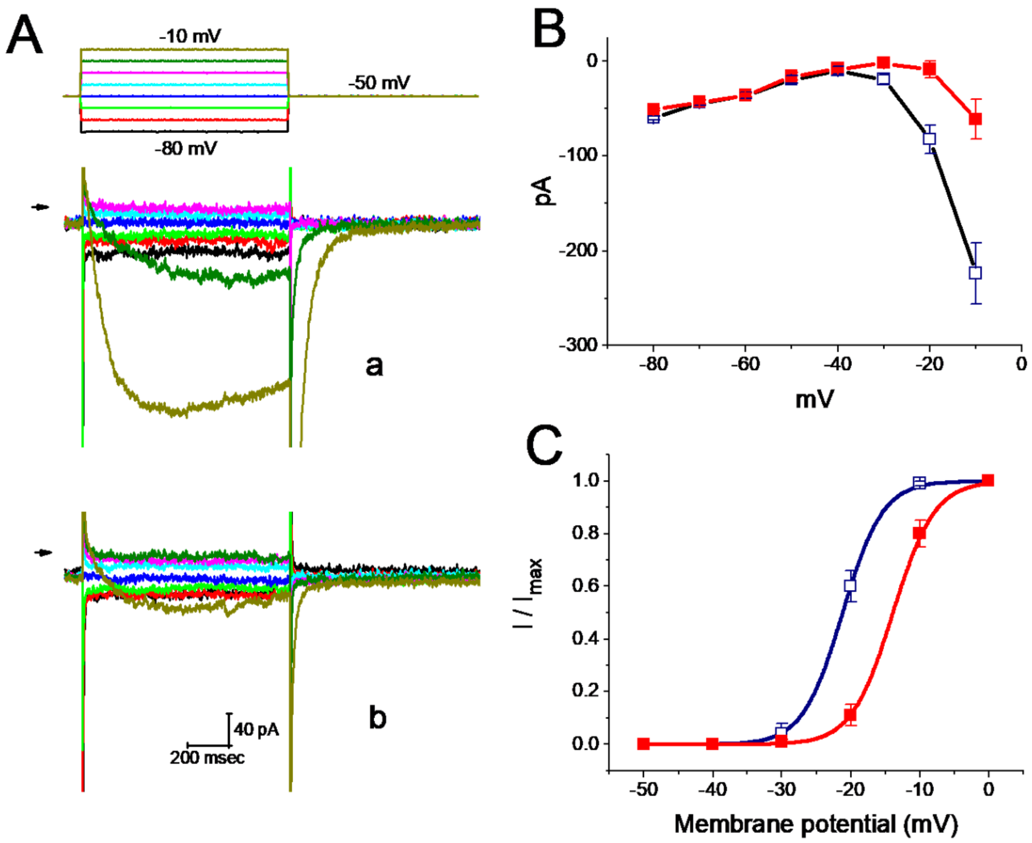


**Figure S3.** Effect of LAP on *I-V* relation of $I_{K(S)}$ recorded from H9c2 cells. (**A**) Superimposed current traces obtained in the absence (**a**) and presence (**b**) of 3 μM LAP. (**B**) Effect of LAP on averaged *I-V* relation of $I_{K(S)}$ in H9c2 cells ($n$ = 10 for each point). □: control; ■: 3 μM LAP. (**C**) Voltage dependence of $I_{K(S)}$ in the absence (□) and presence (■) of 3 μM LAP ($n$ = 9 for each point).

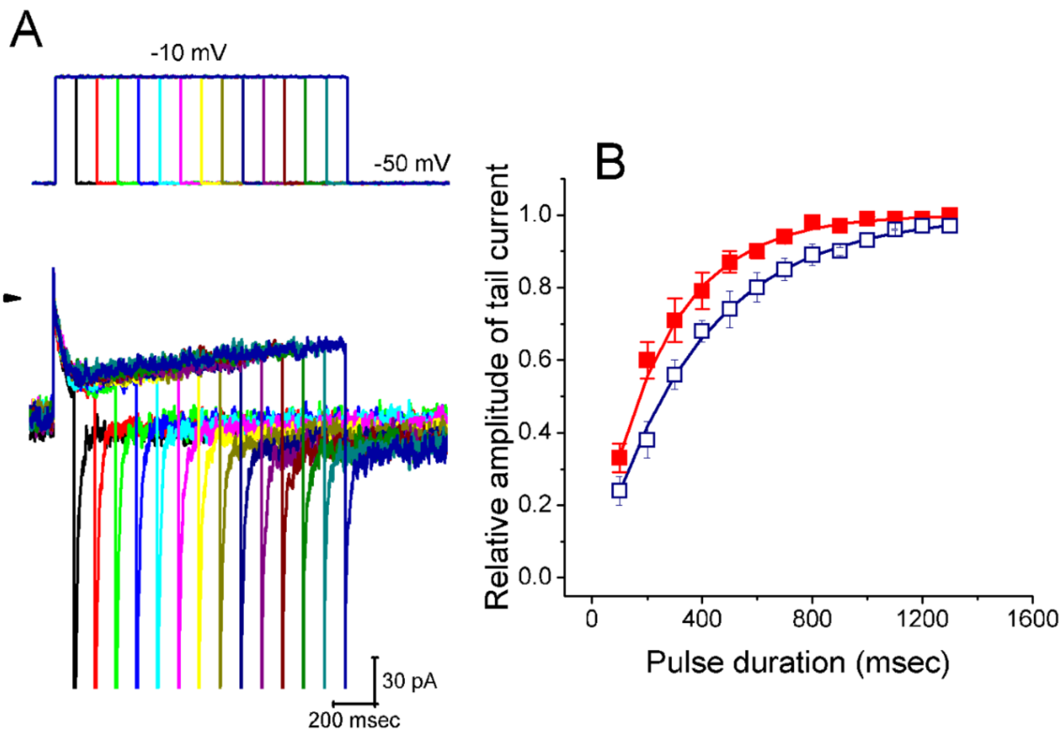


**Figure S4.** Effect of LAP on the recovery of $I_{K(S)}$ deactivation recorded from H9c2 cells. (**A**) Superimposed voltage and current traces obtained in the presence of 3 μM LAP are shown in the upper and lower part of panel, respectively. Arrowhead indicates the zero current level. (**B**) Relationships of pulse intervals versus amplitude of deactivating tail current in the absence (□) and presence (■) of 3 μM LAP (n=7–8 for each point).

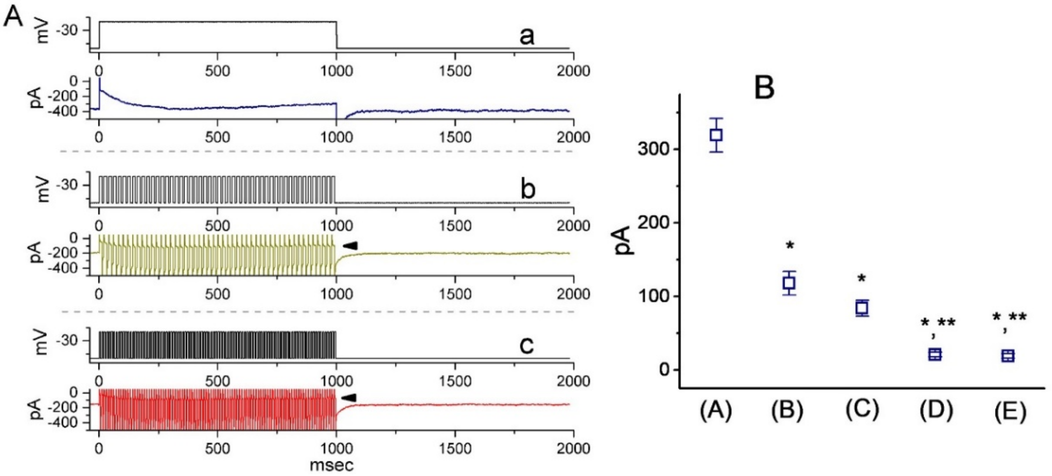


**Figure S5.** Effect of LAP on $I_{K(S)}$ evoked by repetitive stimuli in H9c2 cells. (**A**) Original current traces elicited in response to a single depolarizing pulse (**a**), during repetitive depolarizations with a duration of 12 msec at a rate of 50 Hz (**b**) and that with a duration of 6 msec at a rate of 100 Hz (c). (**B**) Summary of the data showing effect of LAP on $I_{K(S)}$ elicited by a single pulse and repetitive stimuli with 50 or 100 Hz ($n$ = 7–9 for each point). A: single pulse; B: repetitive stimuli with 50 Hz; C: repetitive stimuli with 100 Hz; D: 50-Hz repetitive stimuli with 3 μM LAP; E: 100-Hz repetitive stimuli with 3 μM LAP. *Significantly different from control (i.e., during single pulse; $p < 0.05$) and **significantly

different from the group with repetitive stimuli at 100 Hz alone, $p < 0.05$ by contrasts from one-way analysis of variance (ANOVA).
